# The inflammatory response seen when human omental adipose tissue explants are incubated in primary culture is not dependent upon albumin and is primarily in the nonfat cells

**DOI:** 10.1186/1476-9255-7-4

**Published:** 2010-01-21

**Authors:** John N Fain, Paramjeet Cheema, David S Tichansky, Atul K Madan

**Affiliations:** 1 Department of Molecular Sciences, College of Medicine, University of Tennessee Health Science Center, Memphis, TN 38163, USA; 2 Department of Surgery, College of Medicine, University of Tennessee Health Science Center, Memphis, TN 38163, USA

## Abstract

**Background:**

The present studies were designed to investigate the changes in gene expression during in vitro incubation of human visceral omental adipose tissue explants as well as fat cells and nonfat cells derived from omental fat.

**Methods:**

Adipose tissue was obtained from extremely obese women undergoing bariatric surgery. Explants of the tissue as well as fat cells and the nonfat cells derived by digestion with collagenase were incubated for 20 minutes to 48 h. The expression of interleukin 1β [IL-1β], tumor necrosis factor α [TNFα], interleukin 8 [IL-8], NFκB_1_p50 subunit, hypoxia-inducible factor 1α [HIF1α], omentin/intelectin, and 11β-hydroxysteroid dehydrogenase 1 [11β-HSD1] mRNA were measured by qPCR as well as the release of IL-8 and TNFα.

**Results:**

There was an inflammatory response at 2 h in explants of omental adipose tissue that was reduced but not abolished in the absence of albumin from the incubation buffer for IL-8, IL-1β and TNFα. There was also an inflammatory response with regard to upregulation of HIF1α and NFκB1 gene expression that was unaffected whether albumin was present or absent from the medium. In the nonfat cells derived by a 2 h collagenase digestion of omental fat there was an inflammatory response comparable but not greater than that seen in tissue. The exception was HIF1α where the marked increase in gene expression was primarily seen in intact tissue. The inflammatory response was not seen with respect to omentin/intelectin. Over a subsequent 48 h incubation there was a marked increase in IL-8 mRNA expression and IL-8 release in adipose tissue explants that was also seen to the same extent in the nonfat cells incubated in the absence of fat cells.

**Conclusion:**

The marked inflammatory response seen when human omental adipose tissue is incubated in vitro is reduced but not abolished in the presence of albumin with respect to IL-1β, TNFα, IL-8, and is primarily in the nonfat cells of adipose tissue.

## Background

There is increasing evidence that in central obesity of humans, it is the increase in visceral omental rather than abdominal subcutaneous adipose tissue that best correlates with measures of insulin resistance [1] and cardiovascular disease [2-4]. Furthermore, obesity is associated with a mild inflammatory response in omental adipose tissue [5-7] and inflammation has been considered the link between diabetes and obesity [8,9]. The deleterious effects of obesity with regard to the development of hypertension and type 2 diabetes are primarily seen in extremely obese humans and corrected by weight loss surgery [10-12]. Furthermore the reduction in morbidity due to weight loss surgery has been attributed to a reduction of inflammatory mediators [12].

One model system for studying the inflammatory response is the in vitro incubation of explants of omental adipose tissue from extremely obese humans for 48 h. IL-8 is a chemokine/adipokine whose circulating level is elevated in obese humans [13,14]. More IL-8 is released by adipose tissue explants or adipocytes over 4 h incubation than any other adipokine [15]. Fain et al. [16] reported that in human adipose tissue there is a marked up-regulation of IL-6 or IL-8 mRNA as well as release of IL-6 and IL-8 over a 5 h incubation of explants. The up-regulation of IL-8 mRNA was seen within 3 h and about half of this increase was abolished by blocking the effects of endogenous TNFα and IL-1β [16]. Up-regulation of IL-6 is also seen when freshly isolated rodent adipocytes are incubated in vitro and attributed to effects of collagenase digestion [17]. However, most of the increase in IL-6 and IL-8 mRNA is seen in the cells, other than fat cells, present in human adipose tissue and seen to the same extent in cut pieces of tissue as in the fractions obtained by collagenase digestion [16]. Because IL-8 is a chemokine that could play a major role in recruitment of monocytes into adipose tissue [14] and because of the evidence that TNFα and IL-1β regulated its release by human fat [16] we focused on these adipokines.

The present studies were designed to utilize fat cells and nonfat cells derived from omental adipose tissue as well as omental fat explants obtained from extremely obese women. The three major aims were to investigate [a] the influence of albumin on the inflammatory response in omental adipose tissue explants, [b] whether the up-regulation is in fat cells or the nonfat cells of omental fat and [c] whether co-incubation of nonfat cells with fat cells, both derived from omental adipose tissue, affected their inflammatory response.

## Methods

Visceral omental adipose tissue was obtained from obese women undergoing laparoscopic gastric bypass with Roux-en-y gastroenterostomy surgery for the treatment of extreme obesity in a clinical practice setting. The average body mass index [BMI] of the women whose fat was used for these experiments was 46.0, the age was 43.4 and the blood glucose was 5.4 mM. Each experimental replication involved tissue from a separate individual. Approximately one-third were taking anti-hypertensive agents and another third drugs for diabetes, but were fairly well controlled since the mean plasma glucose was 5.4 mM. The study had the approval of the local IRB and all patients involved gave their informed consent.

The adipose tissue was transported to the laboratory within 15-30 minutes of its removal from the donor. The handling of tissue and cells was done under aseptic conditions. The tissue was cut with scissors into small pieces (5-10 mg) and incubated in buffer [3 ml/g of tissue] for approximately 2-5 min to reduce contamination of the tissue with blood cells and soluble factors. The tissue explants were then centrifuged for 30 sec at 400-× g to remove blood cells and pieces of tissue containing insufficient fat cells to float.

Fat and nonfat cells were isolated by incubating 1.0 g of cut adipose tissue in 2 ml of incubation medium containing 1.3 mg of collagenase in a rotary water bath shaker [100 rpm] for two hours. The collagenase preparation was isolated from Clostridium *histolyticum *(Type 1) and obtained from Worthington Biochemical Corporation of Lakewood, NJ (lot CLS1-4197-MOB3773-B, 219 U/mg). The collagenase digest was then separated from undigested tissue by filtration through 200 μm mesh fabric. Five ml of medium was then added back to the digestion tubes and used to wash the undigested matrix on the filter mesh. This wash solution was combined with the collagenase digest and stromovascular [SV] cells were separated from fat cells and medium by centrifugation in 15 ml tubes for 1 min at 400-× g. The SV cells and fat cells were each suspended in 5 ml of fresh buffer and centrifuged for 10 sec at 400-× g. This medium was removed. The undigested tissue retained on the nylon mesh and the SV cells were combined to obtain the nonfat cells. One gram of adipose tissue explants, the nonfat cell fractions or fat cells obtained by digestion of 1 g of tissue were incubated in a volume of 5 ml for the indicated times. The average diameter of the isolated omental fat cells was 107 microns.

The buffer ordinarily used for incubation of adipose tissue was Dulbecco's modified Eagle's medium/Ham's F12 (1:1, Sigma-Aldrich No. 2906) containing 17.5 mM of glucose, 121 mM of NaCl, 4 mM of KCl, 1 mM of CaCl_2_, 25 mM of HEPES, 22 mM of sodium bicarbonate, 10 mg/ml of defatted bovine serum albumin [unless otherwise stated], 90 μg/ml of penicillin G, 150 μg/ml of streptomycin sulfate and 55 μM of ascorbic acid. The pH of the buffer was adjusted to 7.4 and the buffer filtered through a 0.2 μm filter. IL-8 and TNFα release to the medium was determined using ELISA assays with Duoset reagents from R & D Systems of Minneapolis, MN. Defatted bovine serum albumin powder prepared by heat treatment of serum plus organic solvent precipitation (Bovuminar, containing <0.05 moles of fatty acid/mole of albumin) was obtained from Intergen (Purchase, NY). The low endotoxin bovine albumin was prepared by a similar procedure [#A2934] and obtained from Sigma-Aldrich of St. Louis, MO.

For studies involving mRNA isolation, the nonfat cells, fat cells or tissue were separated from the medium and RNA extracted by Polytron homogenization as described by Chomczynski and Sacchi [18] using 5 ml of a monophasic solution of phenol and guanidine isothiocyanate [Trlzol reagent from Invitrogen of Carlsbad, CA]. The extracts were then spun at 12,000-× g for 10 minutes at 2 to 8°C to separate the fat from the extract.

The assay of mRNA involved real-time qPCR [19,20]. The cDNA was prepared using the Transcriptor First Strand cDNA synthesis Kit from Roche Diagnostics. The quantification of mRNA was accomplished using the Roche Lightcycler 480 Real-time RT-PCR system and their Universal Probe Library of short hydrolysis Locked Nucleic Acid [LNA] dual hybridization probes in combination with the primers suggested by their web-based assay design center http://www.universalprobelibrary.com. Integrated DNA Technologies of Coralville, IA, synthesized the primers. In each assay 70 ng per tube of total RNA [determined by absorption at 260 nm in a spectrophotometer] was used and the ratio of the right to left primers was 1 for each assay. The data were obtained as crossing point values [Cp] obtained by the second derivative maximum procedure as described by Roche Applied Science technical notes LC10/2000 and 13/2001 http://www.roche-applied-science.com/sis/rtpcr/htc/index.jsp. The Cp values are comparable to crossing threshold [Ct] values as defined by ABI or quantification cycle [Cq] http://www.rdml.org. Samples with higher copy number of cDNA have lower Cp values, while those with lower copy numbers have the reverse.

The data were normalized by either the use of cyclophilin mRNA as the recovery standard/calibrator/reference gene or total RNA concentration as recommended by Bustin [21]. The Cp values for cyclophilin A were the same in the nonfat cells as in the fat cells derived from omental adipose tissue [Cp = 28.9 ± 0.3 as the mean ± sem with n of 41 for nonfat cells and 28.5 ± 0.4 for fat cells] while that in unincubated omental adipose tissue was 29.0 [19]. However, over a 24 or 48 h incubation there were significant increases [2.1× at 48 h] in cyclophilin A, so for time course studies the absolute Cp values were used [21]. In this case the ratios were calculated from the ΔCp between unincubated tissue and tissue incubated for a particular time. Relative quantification of the data was calculated using the comparative Cp method, which eliminates the need for standard curves. The arithmetic formula to calculate ratios from ΔCp is based on a log_2 _scale [2^-ΔCp^]. This method is identical to the Comparative C_T _procedure described in the ABI PRISM 7700 Sequence Detection System user Bulletin #2 for quantitative RT-PCR. The calculation of ratios was done without an efficiency correction by assuming that the number of target molecules doubles with every PCR cycle. Caution should be used in comparison of the Cp values between different genes because of the relative efficiencies of the particular primers and probes used for each gene may be different.

A two-tailed Student t-test was used to determine whether differences were significant at a P-value of < 0.05. Statistical analysis of mRNA values was based on the ΔCp values before log_2 _transformation to ratios.

## Results

### The up-regulation of IL-8 release was rapid in onset and accompanied by increases in IL-1β, TNFα, NFκB1, and HIF-1α gene expression

**Figure 1:**
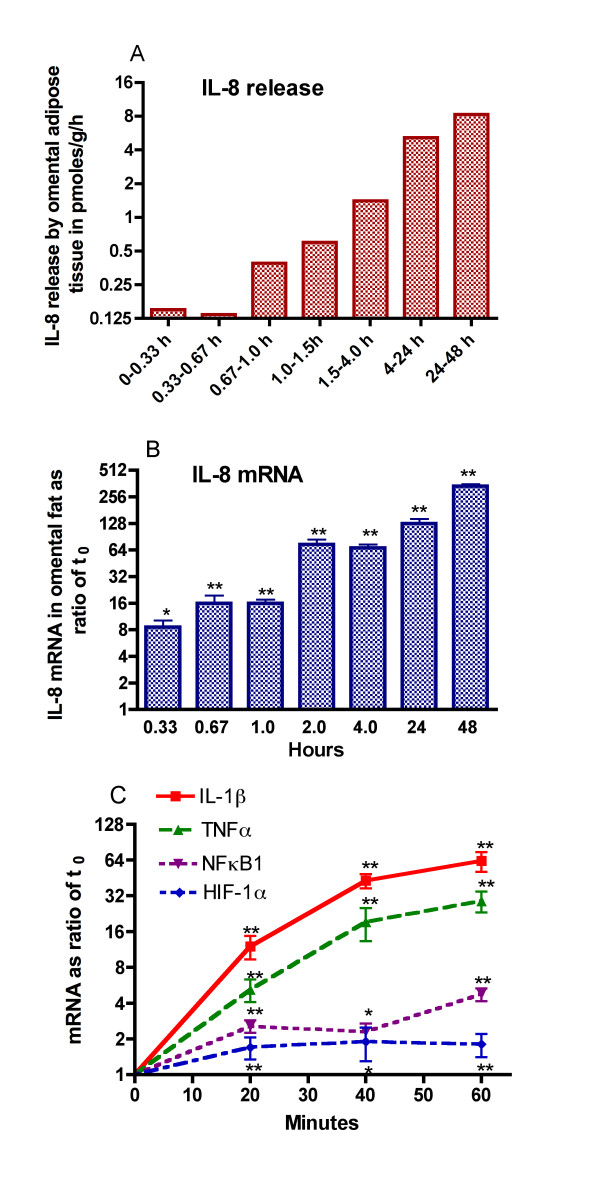
**Upregulation of the inflammatory response is rapid in onset**. Explants of human omental adipose tissue were incubated for the indicated times and samples were taken from the medium to examine IL-8 release. The values in panel A are the means of two experiments. The values for IL-8 mRNA [log_2 _scale] in panel B are the means ± SEM of the ratios of mRNA at the indicated times to that at the start of the incubation for 5 experiments from as many different individuals. The values in panels B & C are based on the changes in absolute Cp values over time as compared to the Cp value in the unincubated tissue. Statistically significant changes in mRNA are indicated as follows: * P < 0.05 and ** P < 0.025. The IL-8 mRNA values at 1 h and all later times shown in panel B were statistically significant from the value at 0.33 h: P < 0.05. The values in panel C for IL-8, IL-1β, TNFα, NFκB1 and HIF-1α mRNA are from a different series of 4 experiments.

The experiments shown in Figure 1 were designed to see how rapid was the upregulation of IL-8 mRNA and protein release by explants of human omental adipose tissue as well as compare IL-8 gene expression at early time points to that of IL-1β, TNFα, NFκB_1 _[p50 subunit], and HIF-1α mRNA. There was a 8-fold increase in IL-8 gene expression after only 20 minutes incubation of omental adipose tissue explants [Figure 1]. By 2 h, there was a 64-fold increase in IL-8 mRNA. The increase in IL-8 was sustained and reached its highest level by 48 h. The release of IL-8 was also upregulated during incubation, but was not seen until after 40 minutes of incubation and further increases were seen over 48 h. The data in figure 1 also indicate that there were similar increases in IL-1β, TNFα, NFκB_1 _[p50 subunit], and HIF-1α mRNA within 20 minutes but the increases in the latter two genes were of lesser magnitude.

### The up-regulation of IL-8 release and mRNA was reduced but not abolished in the absence of albumin

Schlesinger et al [22] reported that albumin enhanced adipokine secretion by human adipocytes. Since our buffer ordinarily contains 1% albumin to bind fatty acids, as is usually done in studies involving fat cells and tissue [23], we compared the inflammatory response of explants of omental adipose tissue with regard to expression of IL-8, IL-1β, TNFα, HIF-1α and NFκB_1 _at 2 h in the presence and absence of albumin [Table 1]. In the absence of albumin, the increases in the mRNAs for IL-1β, TNFα and IL-8 were reduced, but not abolished. However, the 2.4 and 3.5-fold increases in HIF 1α and NFκB_1 _[p50], respectively, seen at 2 h were unaffected by albumin. These increases were statistically significant [p < 0.025]. We include data for omentin/intelectin, whose mRNA, like that of the inflammatory cytokines [19], is primarily found in the nonfat cells of omental adipose tissue [20], as a negative control to demonstrate that not all genes are up-regulated by in vitro incubation of fat for 2 h.

**Table 1:** Effect of albumin on the changes in gene expression over a 2 h incubation

mRNA	Fold change over 2 h	% change in the presence of 1% albumin
IL-8	87 ± 8***	+540 ± 44%***
IL-1β	24 ± 4***	+6000 ± 60%***
TNFα	6.1 ± 0.9***	+920 ± 50%***
HIF-1α	2.4 ± 0.8**	-10 ± 20%
NFKB_1 _[p50]	3.5 ± 0.9**	+40 ± 18%
Omentin/intelectin	0 ± 1	0 ± 20%

Explants of human omental adipose tissue were incubated for 2 h in buffer without or with 1% albumin. The basal data are shown as the mean ± SEM for eight experiments of the ratio of each mRNA at 2 h to that at the start of the incubation. The effects of the albumin are the mean ± SEM of the paired percentage differences. Significant effects of the 2-h incubation and of albumin or serum are indicated as follows: * P < 0.05, ** P < 0.025 and *** P < 0.01

**Figure 2:**
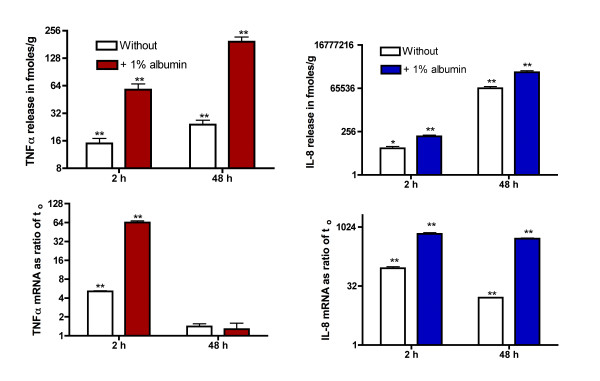
**Incubation of omental fat explants in the absence of albumin reduces but does not abolish upregulation of IL-8 or TNFα mRNA and release**. Explants of human omental adipose tissue were incubated for 2 or 48 h in the absence or presence of 1% albumin. The data are depicted on a log_2 _scale and are the mean ± sem of 4 experiments. The values for mRNA are based on the changes in absolute Cp values over time as compared to the Cp value in the unincubated tissue. Statistically significant changes with time are indicated as follows: * P < 0.05 and ** P < 0.025. The differences without vs. with albumin at 2 and 48 h were significant [P < 0.025] except for TNFα mRNA at 48 h.

The release of IL-8 and TNFα as well as their mRNAs were also enhanced in the presence of albumin as measured at 2 or 48 h but there was still appreciable up-regulation of release in the absence of albumin. If the release of IL-8 had continued over 48 h at the same rate as during the first 40 minutes of incubation [Figure 1], the total release over 48 h would have been less than 7,000 fmoles/g, which was 11% of that observed in the absence of albumin [Figure 2]. The data for IL-8 are expressed in fmoles/g to illustrate that while the release of TNFα over the first 2 h was about 50% of that for IL-8 over 48 h it was less than 0.04% of that for IL-8 [Figure 2].

In another series of experiments using explants of omental adipose tissue, IL-8 mRNA was elevated by 14-fold in the absence of albumin, 184-fold in the presence of 1% endotoxin-free albumin and 343-fold in the presence of 1% bovine albumin as the means of two separate experiments after 48 h. In the same experiments, IL-8 release over 48 h was 4.9-fold greater in the presence of 1% endotoxin-free albumin and 5.4-fold greater in the presence of 1% bovine albumin [data not shown]. Clearly, the effect of albumin is not due to the presence of endotoxin.

### The up-regulation of IL-1β, TNFα, IL-8, and NFκB1 mRNAs is primarily in nonfat cells derived from omental fat

**Figure 3:**
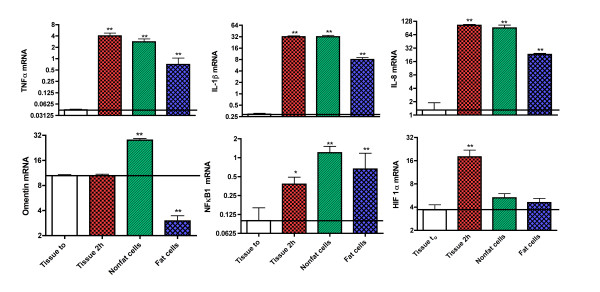
**The inflammatory response in incubated human omental adipose tissue is primarily in nonfat cells and independent of collagenase digestion**. Explants of human omental adipose tissue were taken for mRNA extraction either at the start or end of 2 h incubation while the values for fat cells and nonfat cells were obtained after a 2 h incubation of adipose tissue with collagenase. The values are based on 6-8 experiments from as many different individuals and shown as the mean ± SEM of the ratios of mRNA relative to that of cyclophilin A [log_2 _scale]. Statistically significant changes in tissue samples at 2 h, fat cell and nonfat cells as compared to unincubated tissue (to) are indicated as follows: * P < 0.05 and ** P < 0.025. The differences between fat cells and non-fat cells were statistically significant (P < 0.05) for TNFα, IL-1β, IL-8 and omentin.

The next series of experiments were designed to see whether the enhanced gene expression of IL-1β, TNFα, and IL-8 was in the fat cells the nonfat cells or both. Because of the rapid up-regulation of inflammatory genes in studies comparing the response in fat cells and nonfat cells, it was necessary to use tissue controls incubated for the length of time required for collagenase digestion of adipose tissue. The data in Figure 3 demonstrate that the increases in the mRNAs for IL-1β, TNFα, NFκB_1 _and IL-8 were far higher in nonfat than in fat cells isolated from adipose tissue after 2 h incubation with collagenase. These differences were statistically significant with a P < 0.025. Furthermore, the expression of the mRNAs for IL-1β, TNFα, and IL-8 in nonfat cells was equivalent to that in intact tissue incubated for the same period of time without collagenase.

However, for HIF1α there was no significant increase in its gene expression in either fat cells or nonfat cells while there was in tissue incubated for 2 h. This was in contrast to NFκB_1 _whose gene expression was significantly elevated in tissue, fat cells and nonfat cells to about the same extent. Data for omentin/intelectin are included in figure 3 as a control, because it is a gene whose expression is not up-regulated over a 2 h incubation [Table 1] and is primarily expressed in the nonfat cells of adipose tissue [15].

**Figure 4:**
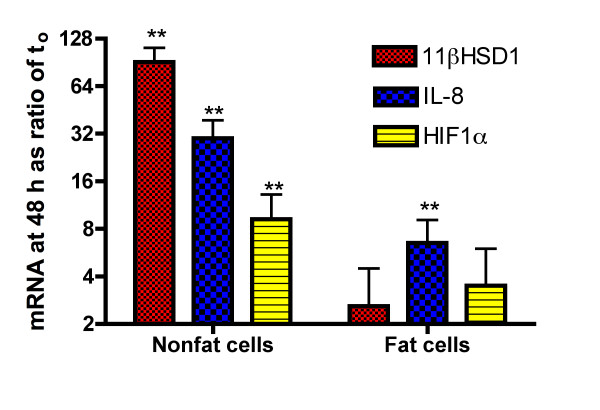
**Comparison of IL-8, HIF 1α, and 11βHSD1 upregulation in fat cells vs. nonfat cells incubated 48 h**. The nonfat cells and fat cells, obtained by digestion of human adipose tissue with collagenase, were incubated for 48 h. The values shown are the mean ± SEM of the ratios of mRNA at 48 h as compared to that at the start of the incubation [log_2 _scale] for 4 experiments from as many different individuals. Statistically significant changes are indicated as follows: * P < 0.05 and ** P < 0.025.

The question of what happens when isolated fat cells or nonfat cells are incubated in vitro for 48 h was examined in the studies shown in figure 4. There were significant additional increases in the mRNAs for IL-8, HIF1α, and 11β HSD1 in the nonfat cells over the 48 h incubation. There was also a significant increase in IL-8 gene expression in isolated fat cells that was about 22% of that seen in the nonfat cells. In contrast, there was no increase in HIF-1α or 11β HSD1 gene expression in fat cells. The initial ratios of IL-8 and HIF1α in nonfat cells to fat cells was 9.2 and 4.6-× while that of 11β HSD1 was 0.25 indicating that there is 4-fold more 11β HSD1 in fat cells than in nonfat cells. Interestingly over the 48 h incubation there was a marked increase in 11β HSD-1 gene expression in nonfat but not in fat cells [Figure 4].

### The increases in IL-8 release and mRNA in non-fat cells during incubation are unaltered by the presence of fat cells

**Figure 5:**
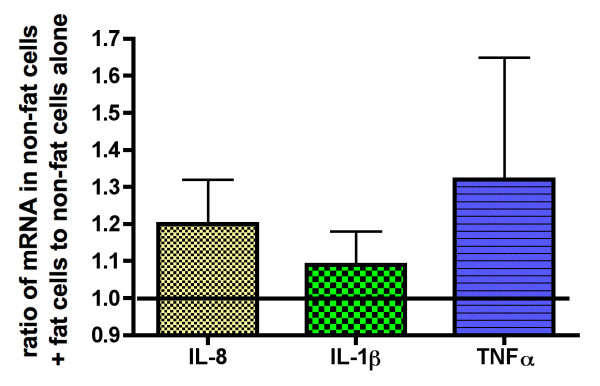
**Incubation of fat cells with the nonfat cells does not significantly affect upregulation of IL-8, IL-1β or TNFα mRNA**. The nonfat cells obtained by incubation of human adipose tissue with collagenase was incubated for 48 h either without or with the fat cells obtained from the same amount of tissue. The values are the mean ± SEM of the paired differences for 8 experiments from as many different individuals and shown as the ratio of mRNA in nonfat cells plus fat cells to that in nonfat cells. None of the differences were statistically significant with a P < 0.05.

These studies were designed to determine whether the upregulation of IL-8 mRNA as well as its release were stimulated or inhibited by the concurrent presence of fat cells [Table 2]. The release of IL-8 over 48 h and the mRNA content at 48 was the same in nonfat cells as in tissue explants incubated for the same amount of time. Another approach to examining the possible role of factors released by fat cells on upregulation of the inflammatory response in non-fat cells is the co-incubation of fat cells with non-fat cells. There was no significant increase in up-regulation of IL-8, IL-1β or TNFα mRNA over a 48 h incubation of nonfat cells with the fat cells derived from the same amount of tissue [Figure 5].

**Table 2:** Fat cells are not required for the up-regulation of IL-8 release and IL-8 mRNA seen in nonfat cells over a 48 h incubation

IL-8 mRNA	Change in tissue after 48 h [ratio]	% Change in nonfat cells incubated for 48 h as compared to tissue	% Change in nonfat cells isolated after 48 h as compared to tissue
	832-X	+38 ± 17%	+3 ± 12%
IL-8 release	48 h release by tissue in pmoles/g	% Change in nonfat cells incubated for 48 h as compared to tissue	
	1450	+1 ± 16%	

The change in IL-8 mRNA at 48 h is the fold-change derived from the ΔCp over 48 h of -9.6 ± 0.3. The values are from 8 experiments and the % changes are the mean ± SEM of the paired differences. None were statistically significant with a P < 0.05.

## Discussion

In mice, given enough lipopolysaccharide to kill 40% of the mice by 24 h, increases in MCP-1, IL-6, nerve growth factor, TNFα and HIF-1α were seen in adipose tissue within 4 h [24]. Furthermore, there was a marked increase in HIF1α protein accompanied by even greater changes in mRNA [24]. It is unclear how endotoxin elevates HIF-1α in the fat of mice and this could be independent of hypoxia. We observed a similar rapid increase in HIF1α and NFκB_1 _expression simply by incubating human adipose tissue explants in vitro.

While albumin enhanced the release of IL-8 and its gene expression, it did not affect the early increase in the inflammatory response as judged by increases in expression of HIF1α or NFκB1. Furthermore albumin effects were primarily due to factors other than endotoxin contamination, which is in agreement with the findings of Schlesinger et al [22]. These investigators found that while 2% bovine albumin, but not 0.7%, significantly stimulated the release of IL-6, IL-8 and TNFα by freshly isolated human adipocytes. However, albumin had much greater effects on in vitro differentiated human adipocytes [22]. Exactly what accounts for the effects of albumin is unclear but albumin is able to bind many non-polar molecules and can bind up to 7 moles of fatty acid per mole of albumin [25]. The albumin we used was isolated by a heat-shock process in the presence of octanoic acid resulting is a low fatty acid content, less than 0.05 moles/mole, but it is unclear whether this small amount of fatty acid can account for the effects. Traditionally adipose tissue or fat cells are incubated in the presence of 1 to 4% albumin to bind fatty acids released during lipolysis [23]. This is done because lipolysis by rat fat cells is inhibited in the absence of albumin to bind fatty acids released during lipolysis [26]. Albumin has been shown to influence inducible nitric oxide synthase in macrophage and smooth muscle cells [27] and induce an inflammatory response in proximal tubular cells [28]. While what is responsible for the inflammatory effect of albumin remains to be established, it had no effect of the increases in HIF1α or NFκB1 expression suggesting that albumin effects are exerted at a step between their activation and that of enhanced IL-8, TNFα, and IL1-β gene expression.

Whether the inflammatory response seen when adipose tissue is incubated in vitro is due to relative hypoxia secondary to cutting the blood supply remains to be established. Trayhurn et al [29] have emphasized the pervasive effects of hypoxia on the inflammatory response of adipose tissue in obesity. The present results are compatible with this hypothesis as an explanation for the inflammatory response seen when human omental fat explants are incubated in vitro. The effects of hypoxia in tissues appear to be mediated in part through HIF1α, which is a major transcription factor that responds to hypoxia [6,29]. While initial studies on the role of HIF1α suggested that activation was primarily translational control of its proteolytic degradation, more recently HIF1α gene activation has been shown to play a role [30]. Hypoxia activates other transcription factors and one of them is NFκB_1_, which is also what we observed in human adipose tissue. The gene expression of both HIF1α and NFκB1 was elevated after only a 20 minute incubation of adipose tissue but at that time other inflammatory response genes were also activated making it impossible to determine a causal relationship. The finding that HIF1α mRNA up-regulation was far greater in intact adipose tissue explants than in nonfat cells or isolated fat cells suggests that incubated tissue is a more hypoxic environment. However, we did not measure HIF1α protein whose altered rate of degradation in the presence of hypoxia is the primary regulator of the inflammatory response.

The 9-fold up-regulation of HIF-1α mRNA over a 48 h incubation of nonfat cells isolated from omental adipose tissue is comparable to what Gesta et al [31] reported using explants of human subcutaneous adipose tissue. They suggested that this was due to the relative hypoxia of tissue explants and accounted for the increase in TNFα mRNA. For reasons that are unclear, they found a different time course for TNFα in that the maximal increase in TNFα mRNA was seen at 48 h while we previously reported an increase that was maximal at 4 h and declined over the next 44 h [32].

The inflammatory response as measured by accumulation of IL-1β, TNFα and IL-8 mRNAs was seen in both fat and nonfat cells of human omental fat. However, the increases in the nonfat cells for IL-1β, TNFα and IL-8 obtained after 2 h isolation procedure were identical to those seen when intact tissue was incubated for the same amount of time. This indicates that collagenase digestion is not responsible for the up-regulation as initially suggested by Ruan et al [17]. The expression of IL-1β, TNFα and IL-8 was rather less in fat cells than was seen in the nonfat cells in agreement with studies on the release of TNFα and IL-6 over a 4 h incubation where the nonfat cells accounted for over 90% of total release [32].

The role of fat cells as primary triggers for the inflammatory response in nonfat cells of adipose tissue could not be determined during the first 2 hours of incubation because it took that long to separate fat cells from nonfat cells. However, we found that the subsequent incubation of the nonfat cells with the fat cells for 48 h had no effect on up-regulation in fat cells. If there is paracrine cross-talk between fat cells and nonfat cells, it clearly has little influence upon the inflammatory response with respect to IL-8 since it was seen to the same extent in isolated fat cells, isolated nonfat cells or intact tissue explants. But we cannot exclude the importance of paracrine interactions between fat cells and the nonfat cells of omental adipose tissue prior to the start of the incubation during the time required to isolated the fat cells by digestion with collagenase. Our data also do not exclude cross talk between factors released by macrophages and other cells in the nonfat cell fraction.

One finding of interest was that while 11β-HSD1, which is initially enriched in fat cells by 4-fold, is up-regulated over 48 h by 9-fold in the nonfat cells but not in the fat cells. 11β-HSD1 is thought to be involved in the conversion of cortisone to cortisol and elevated levels of cortisol are associated with hypertension and insulin resistance [33]. Furthermore, 11β-HSD1 gene expression is enhanced in visceral obesity [34], which could contribute to insulin resistance by enhancing local conversion of cortisone to cortisol [33,34].

It should be noted that all the data were obtained with samples of omental adipose tissue from extremely obese women and whether the findings are applicable to fat from men and/or non-obese women remains to be established. Furthermore, the protein levels may not correlate as well with gene expression levels as they did with IL-8 and TNFα.

There is a growing consensus that massive obesity is accompanied by an inflammatory response in adipose tissue [5-12] and that this is primarily due to visceral obesity [4]. This can be mimicked in vitro by incubating explants of human omental fat from severely obese women and results in a rapid inflammatory response that can be seen within 20 minutes with respect to gene expression of inflammatory response proteins such as HIF1α and NFκB as well as inflammatory adipokines such as TNFα and IL-1β, and IL-8. Enhanced release of IL-8 could be seen after a 40-minute lag period and the present results provide further support for the hypothesis that this primarily occurs in the nonfat cells. Exactly what it is about obesity that induces an inflammatory response in vivo is unclear but may well relate to relative hypoxia for the large fat cells. The initial trigger could be breakdown of large fat cells and/or enhanced release of factors such as fatty acids that recruit mononuclear cells into adipose tissue. IL-8 is a chemokine that could well be involved in monocyte recruitment and the accumulation of mononuclear cells in adipose tissue is enhanced in obesity [35,36]. It is probable that the majority of the release of adipokines by nonfat cells in human adipose tissue is due to macrophages and other mononuclear phagocytic cells. These adipokines could account for generalized inflammation secondary to the release of inflammatory factors into the circulation. These factors and/or enhanced release of fatty acids could be responsible for the development of hypertension and diabetes in obesity.

## Conclusions

The up-regulation of the inflammatory response seen when human omental adipose tissue is incubated in vitro is primarily in the nonfat cells of adipose tissue, albumin enhances the up-regulation of adipokines but not of HIF-1α or NFκB_1 _and the up-regulation of the inflammatory response of isolated fat cells or nonfat cells does not appear to be influenced by paracrine cross-talk.

## Competing interests

The authors declare that they have no competing interests.

## Authors' contributions

JF designed the experiments, analyzed the data and drafted the manuscript. PC carried out the laboratory studies and analysis of mRNA. DT and AM selected the donors, obtained the samples of fat and aided in the interpretation of the data. All authors read and approved the final manuscript.

## References

[B1] Piche ME Lapointe A Weisnagel SJ Corneau L Nadeau A Bergeron J Lemieux S Regional body fat distribution and metabolic profile in postmenopausal women Metabolism 2008 57 1101 1107 10.1016/j.metabol.2008.03.015 18640388

[B2] Despres JP Lemieux I Bergeron J Pibarot P Mathieu M Larose E Rodés Cabau J Bertrand OF Poirier P Abdominal obesity and the metabolic syndrome: contribution to global cardiometabolic risk Arterioscler Thromb Vasc Biol 2008 28 1039 1049 10.1161/ATVBAHA.107.159228 18356555

[B3] Canoy D Boekholdt SM Wareham N Luben R Welch A Bingham S Buchan I Day N Khaw KT Body fat distribution and risk of coronary heart disease in men and women in the European prospective investigation into cancer and nutrition in Norfolk cohort: a population-based prospective study Circulation 2007 115 2933 2943 10.1161/CIRCULATIONAHA.106.673756 18071080

[B4] Montague CT O'Rahilly S The perils of portliness: causes and consequences of visceral adiposity Diabetes 2000 49 883 888 10.2337/diabetes.49.6.883 10866038

[B5] Wellen KE Hotamisligil GS Inflammation, stress, and diabetes J Clin Invest 2005 115 1111 1119 15864338 10.1172/JCI25102 PMC1087185

[B6] Trayhurn P Wood IS Adipokines: inflammation and the pleiotropic roles of white adipose tissue Br J Nutr 2004 92 347 355 10.1079/BJN20041213 15469638

[B7] Arner P Introduction: the inflammation orchestra in adipose tissue J Intern Med 2007 262 404 407 10.1111/j.1365-2796.2007.01850.x 17875175

[B8] Dandona P Aljada A Bandyopadhyay A Inflammation: the link between insulin resistance, obesity and diabetes Trends Immunol 2004 25 4 7 10.1016/j.it.2003.10.013 14698276

[B9] Pories WJ Swanson MS MacDonald KG Long SB Morris PG Brown BM Barakat HA deRamon RA Israel G Dolezal JM Who would have thought it? An operation proves to be the most effective therapy for adult-onset diabetes mellitus Ann Surg 1995 222 339 350 10.1097/00000658-199509000-00011 7677463 PMC1234815

[B10] Pories WJ Bariatric surgery: risks and rewards J Clin Endocrinol Metab 2008 93 S89 S96 10.1210/jc.2008-1641 18987275 PMC2729256

[B11] Sugerman HJ Wolfe LG Sica DA Clore JN Diabetes and hypertension in severe obesity and effects of gastric bypass-induced weight loss Ann Surg 2003 237 751 756 10.1097/00000658-200306000-00002 12796570 PMC1514677

[B12] Cottam DR Mattar SG Barinas-Mitchell E Eid G Kuller L Kelley DE Schauer PR The chronic inflammatory hypothesis for the morbidity associated with morbid obesity: implications and effects of weight loss Obes Surg 2004 14 589 600 10.1381/096089204323093345 15186624

[B13] Straczkowski M Dzienis-Straczkowska S Stepien A Kowalska I Szelachowska M Kinalska I Plasma interleukin-8 concentrations are increased in obese subjects and related to fat mass and tumor necrosis factor-α system J Clin Endocrinol Metab 2002 87 4602 4606 10.1210/jc.2002-020135 12364441

[B14] Kim CS Park HS Kawada T Kim JH Lim D Hubbard NE Kwon BS Erickson KL Yu R Circulating levels of MCP-1 and IL-8 are elevated in human obese subjects and associated with obesity-related parameters Int J Obes 2006 30 1347 1355 10.1038/sj.ijo.0803259 16534530

[B15] Fain JN Release of interleukins and other inflammatory cytokines by human adipose tissue is enhanced in obesity and primarily due to the nonfat cells Vitam Horm 2006 74 443 477 full_text 17027526 10.1016/S0083-6729(06)74018-3

[B16] Fain JN Bahouth SW Madan AK Involvement of multiple signaling pathways in the post-bariatric induction of IL-6 and IL-8 mRNA and release in human visceral adipose tissue Biochem Pharmacol 2005 69 1315 1324 10.1016/j.bcp.2005.02.009 15826602

[B17] Ruan H Zarnowski MJ Cushman SW Lodish HF Standard isolation of primary adipose cells from mouse epididymal fat pads induces inflammatory mediators and down-regulates adipocyte genes J Biol Chem 2003 278 47584 47593 10.1074/jbc.M305257200 12975378

[B18] Chomczynski P Sacchi N Single-step method of RNA isolation by acid guanidinium thiocyanate-phenol-chloroform extraction Anal Biochem 1987 152 156 159 10.1016/0003-2697(87)90021-2 2440339

[B19] Fain JN Buehrer B Bahouth SW Tichansky DS Madan AK Comparison of messenger RNA distribution for 60 proteins in fat cells vs the nonfat cells of human omental adipose tissue Metabolism 2008 57 1005 1015 10.1016/j.metabol.2008.02.019 18555844

[B20] Fain JN Sacks HS Buehrer Bahouth SW Garrett E Wolf RY Carter RA Tichansky DS Madan AK Identification of omentin mRNA in human epicardial adipose tissue: comparison to omentin in subcutaneous, internal mammary artery periadventitial and visceral abdominal depots Intl J Obes 2008 32 810 815 10.1038/sj.ijo.0803790 18180782

[B21] Bustin SA Absolute quantification of mRNA using real-time reverse transcription polymerase chain reaction assays J Mol Endocrinol 2000 25 169 193 10.1677/jme.0.0250169 11013345

[B22] Schlesinger JB van Harmelen V Alberti-Huber CE Hauner H Albumin inhibits adipogenesis and stimulates cytokine release from human adipocytes Am J Physiol Cell Physiol 2006 291 C27 C33 10.1152/ajpcell.00172.2005 16452161

[B23] Arner P Techniques for the measurement of white adipose tissue metabolism: a practical guide Int J Obes Relat Metab Disord 1995 19 435 442 8520631

[B24] Leuwer M Welters I Marx G Rushton A Bao H Hunter L Trayhurn P Endotoxaemia leads to major increases in inflammatory adipokine gene expression in white adipose tissue of mice Pflugers Arch - Eur J Physiol 2009 457 731 741 10.1007/s00424-008-0564-8 18677510

[B25] Vusse GJ van der Albumin as fatty acid transporter Drug Metab Pharmacokinet 2009 24 300 307 10.2133/dmpk.24.300 19745557

[B26] Rodbell M Modulation of lipolysis in adipose tissue by fatty acid concentration in fat cell Ann NY Acad Sci 1965 131 302 314 10.1111/j.1749-6632.1965.tb34798.x 4285776

[B27] Poteser M Wakabayashi I Serum albumin induces iNOS expression and NO production in RAW 267.4 macrophages Br J Pharmacol 2004 143 143 151 10.1038/sj.bjp.0705897 15289288 PMC1575263

[B28] Takaya K Koya D Isono M Sugimoto T Sugaya T Kashiwagi A Haneda M Involvement of ERK pathway in albumin-induced MCP-1 expression in mouse proximal tubular cells Am J Physiol Renal Physiol 2003 284 F1037 F1045 12517735 10.1152/ajprenal.00230.2002

[B29] Trayhurn P Wang B Wood IS Hypoxia in adipose tissue: a basis for the dysregulation of tissue function in obesity Br J Nutr 2008 100 227 235 10.1017/S0007114508971282 18397542

[B30] Tai TC Wong-Faull DC Claycomb R Wong DL Hypoxic stress-induced changes in adrenergic function: role of HIF1 α J Neurochem 2009 109 513 524 10.1111/j.1471-4159.2009.05978.x 19220706

[B31] Gesta S Lolmede K Daviaud D Berlan M Bouloumie A Lafontan M Valet P Saulnier-Blache JS Culture of human adipose tissue explants leads to profound alteration of adipocyte gene expression Horm Metab Res 2003 35 158 163 10.1055/s-2003-39070 12734776

[B32] Fain JN Bahouth SW Madan AK TNFα release by the nonfat cells of human adipose tissue Intl J Obes 2004 28 616 622 10.1038/sj.ijo.0802594 14770194

[B33] Walker BR Extra-adrenal regeneration of glucocorticoids by 11β- hydroxysteroid dehydrogenase type 1: physiological regulator and pharmacological target for energy partitioning Proc Nutr Soc 2007 66 1 8 10.1017/S002966510700523X 17343766

[B34] Mariniello B Ronconi V Rilli S Bernante P Boscaro M Mantero F Giacchetti G Adipose tissue 11{β}-hydroxysteroid dehydrogenase type 1 expression in obesity and Cushing's syndrome Eur J Endocrinol 2006 155 435 441 10.1530/eje.1.02228 16914598

[B35] Xu H Barnes GT Yang Q Tan G Yang D Chou CJ Sole J Nichols A Ross JS Tartaglia LA Chen H Chronic inflammation in fat plays a crucial role in the development of obesity-related insulin resistance J Clin Invest 2003 112 1785 1788 14679177 10.1172/JCI19451 PMC296998

[B36] Weisberg SP McCann D Desai M Rosenbaum M Leibel RL Ferrante AW Jr Obesity is associated with macrophage accumulation in adipose tissue J Clin Invest 2003 112 1796 1808 14679176 10.1172/JCI19246 PMC296995

